# Efficacy of prophylactic negative pressure wound therapy after open ventral hernia repair: a systematic review meta-analysis

**DOI:** 10.1186/s12893-023-02280-4

**Published:** 2023-12-11

**Authors:** Yang Xu, Shuai Shao, ZeZhong Gong, HyokJu Ri, ZhaoHui Xu, HaoNan Kang, Yan Shan, Boureima Hamidou Amadou, YanYing Ren, Fan Zhang, Xin Chen

**Affiliations:** 1 Department of Hernia and Colorectal Surgery, The Second Hospital of Dalian Medical University, Dalian, 116023 People’s Republic of China; 2 Department of Colorectal Surgery, the Hospital of Pyongyang Medical College, Pyongyang, 999093 Democratic People’s Republic of Korea

**Keywords:** Ventral hernia, Surgical site occurrences, OVHR, NPWT

## Abstract

**Introduction:**

The susceptibility to surgical site occurrence (SSO) is high following ventral hernia repair (VHR) surgery. SSO severely increases the physical and mental burden on patients. The main purpose of this review was to analyze the efficacy of negative pressure wound therapy (NPWT) after open VHR(OVHR) and explore benefits to patients.

**Methods:**

The Cochrane Library, PubMed, and Embase databases were searched from the date of establishment to 15 October 2022. All randomized controlled trials and retrospective cohort studies comparing NPWT with standard dressings after OVHR were included. The Revman 5.4 software recommended by Cochrane and the STATA16 software were used in this meta-analysis.

**Results:**

Fifteen studies (involving 1666 patients) were identified and included in the meta-analysis, with 821 patients receiving NPWT. Overall, the incidence rate of SSO in the NPWT group was lower compared to the control group (odds ratio [OR] = 0.44; 95% confidence interval [CI] = 0.21–0.93; I^2^ = 86%; *P* = 0.03). The occurrence rate of surgical site infection (SSI; OR = 0.51; 95% CI = 0.38–0.68, *P* < 0.001), wound dehiscence (OR = 0.64; 95% CI = 0. 43–0.96; P = 0.03), and hernia recurrence (OR = 0.51; 95% CI = 0.28–0.91, *P* = 0.02) was also lowered. There was no significant difference in seroma (OR = 0.76; 95% CI = 0.54–1.06; *P* = 0.11), hematoma (OR = 0.53; 95% CI = 0.25–1.11; *P* = 0.09), or skin necrosis (OR = 0.83; 95% CI = 0.47–1.46; *P* = 0.52).

**Conclusion:**

NPWT can effectively decrease the occurrence of SSO, SSI wound dehiscence and hernia recurrence and should be considered following OVHR.

**Supplementary Information:**

The online version contains supplementary material available at 10.1186/s12893-023-02280-4.

## Introduction

Ventral hernia (VH) is a common complication after abdominal surgery. About 10 to 23% of patients develop incisional hernias [1]. The United States of America (USA) spends more than $3.2 billion per year on surgical treatment of adult ventral hernia [2]. Compared to low body mass index (BMI), patients with obesity have a higher incidence of abdominal wall hernias, larger hernia defects, poorer prognosis, and a higher risk of hernia recurrence. Open ventral hernia repair (OVHR) is favorable for large and complex anatomy due to advantages, such as clear anatomy, low operation difficulty, low effect of obesity, short anesthesia time, and low impact on intestinal function [3]. However, the potential dead space and mesh after OVHR can easily lead to surgical site occurrence (SSO), including surgical site infection (SSI), wound dehiscence, seroma, hematoma, skin necrosis, and hernia recurrence [4]. SSIs and other complications may further lead to extended hospital stays, delayed recovery, increased psychological stress, and higher treatment costs [5]. Some factors associated with SSI include weakened immunity, poor nutrition, advanced age, diabetes, use of corticosteroids, and incarcerated hernia. Therefore, breakthrough techniques in surgery are a priority for patients with high-risk factors for SSI.

Negative pressure wound therapy (NPWT) is a widely used wound care technology, mainly for soft tissue trauma and fracture [6]. NPWT includes a drainage system involving the wound dressing and its connected device. By applying negative pressure ranging from − 75 mmHg to − 150 mmHg, any wound and tissue fluid drawn from the area is collected into the vacuum device [7]. Negative pressure suction devices can be used in several ways. Their advantages include removing wound exudates, promoting apposition of the skin edges, changing the microenvironment and stimulating the formation of granulation tissue, and new blood vessel formation [8]. NPWT has been shown to reduce the incidence of complications in various primary closures [9]. However, the efficacy of NPWT after OVHR is unclear. The main purpose of our systematic review is to highlight the effect of prophylactic negative pressure wound therapy (pNPWT) in preventing SSO. The secondary outcome was the incidence rate of other complications.

## Methods

### Data strategy

The Cochrane Central Register of Controlled Trials, MEDLINE, and Embase databases were searched from the respective dates of inception until 15 October 2022. A combination of Mesh and text terms, such as “ventral hernia repair,” “incisional hernia repair,” “VHR,” “abdominal wall reconstruction,” “negative pressure therapy,” “negative pressure wound therapy,” “NPWT,” “VAC,” and “vacuum-assisted closure,” were used. References from related articles, reviews, and meta-analyses were manually searched. This review conformed to the AMSTAR checklist and adhered to the guidelines outlined in the Preferred Reporting Items for Systematic Reviews and Meta-analyses (PRISMA) statement [10].

### Inclusion criteria

This review mainly included randomized controlled trials (RCTs) and retrospective cohort studies comparing pNPWT with standard dressing after OVHR. Only articles with adult patients (> 18 years of age) undergoing OVHR were included in the analysis. The intervention group included pNPWT technique using devices such as PREVENA, PICO, and VAC based on different degrees of negative pressure.

### Exclusion criteria

The excluded articles were those with pNPWT used alone or compared with other technologies; reporting no outcomes; systematic reviews, study protocols, and case reports on the placement of pNPWT in inguinal and perineal hernias; and published in a language other than English.

### Outcomes

SSO was the primary outcome of this review. SSI, seroma, hematoma, wound dehiscence, skin necrosis, and hernia recurrence were the secondary outcomes considered to further analyze the effect on SSO results.

### Data collection and analysis

Two authors independently examined the title and abstract of each article after electronic retrieval. In case of disagreements, a third independent author evaluated the article. The articles that potentially met the inclusion criteria were further analyzed, and only those meeting all inclusion criteria were used for analysis.

### Quality assessment of included article

Cochrane tools were used to assess the RCT bias, including selection bias, performance bias, detection bias, attrition bias, reporting bias and other sources of bias. The Newcastle-Ottawa Cohort Study Quality Assessment Scale was used to assess the risk of cohort study bias. A third independent author decided inclusion in case of disagreements between the two authors.

### Statistical analysis

All statistical analyses were performed using the STATA16 software and Cochrane’s Review Manager 5.4 software. Meta-analysis was performed on the odds ratio (OR) of the binary variables and a *P*-value of < 0.05 was considered statistically significant. The I^2^ statistic was used to assess intergroup heterogeneity. The random-effects model and fixed-effects model were used, according to the heterogeneity among included studies. The results of the meta-analysis were presented graphically as forest plots. Egger’s test was used to assess publication bias according to the number of articles.

## Results

**Fig. 1:**
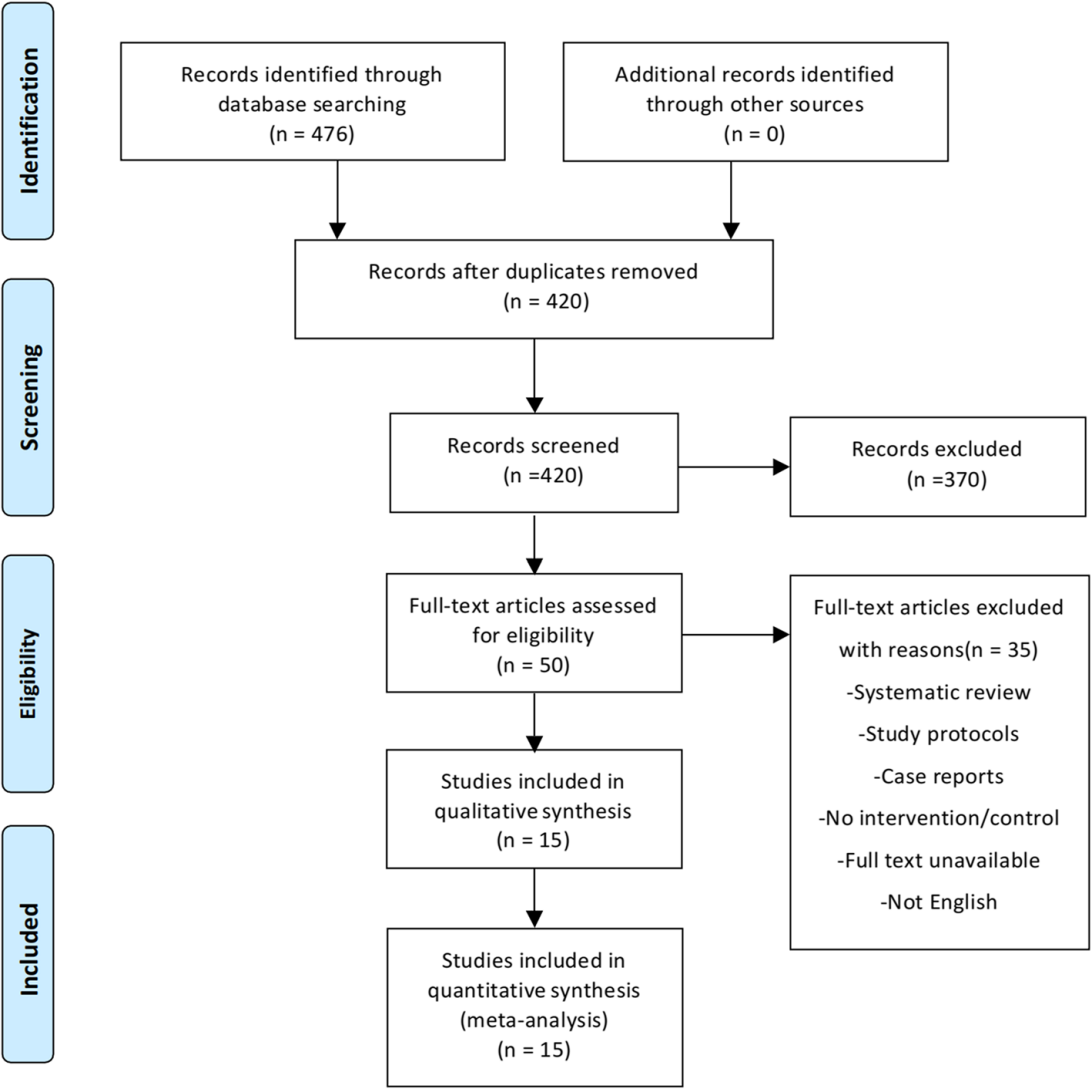
PRISMA diagram showing the screening process

Overall, a total of 476 studies were identified from the databases. Fifty full texts were then shortlisted based on title and abstract analysis, of which 15 articles finally met the inclusion criteria. The specific inclusion process flowchart is shown in Fig. 1. A total of 1666 patients were included in the selected articles, of which 821 patients underwent pNPWT.

The general characteristics of the articles included in the meta-analysis are shown in Table 1. The sample size of these studies ranged from 35 to 180 and the maximum follow-up duration varied from 30 days to 38.5 months. Most of the studies were retrospective cohort analyses and only 2 were RCTs. PREVENA was the most widely used pNPWT device for OVHR. PREVENA was used and described in eight articles. The negative pressure applied to the incision varied from − 75 mmHg to − 125 mmHg.

**Table 1:** General characteristics of the articles included in the meta-analysis

Author (year)	Centre (country)	Study type	Age (NPWT: control)	BMI (NPWT: control)	Sample size (NPWT, n)	Follow-up duration	Details of NPWT	Details of control	Mesh position	Mesh type	Outcomes
Bueno-Lledó et al. (2020) [11]	Single center (Spain)	RCT	51.6:51.3	NR	146 (NPWT, 72)	30 days	PICO, −80 mmHg× 7 days	Standard dressing	Sublay	Synthetic	1) 2) 3) 4) 5)
Conde´-Green et al. (2013) [12]	Single center (USA)	Retrospective	54:55	36.4:36.1	56 (NPWT, 23)	15 months	VAC, −125 mmHg × 5 days	Conventional dry gauze dressing	NR	Biological	1) 3) 4) 5) 6) 7)
de Vries et al. (2017) [13]	Single center (The Netherlands)	Retrospective	58.9:59.7	26.9:27.7	66 (NPWT, 32)	30 days	PREVENA or a home-made wound dressing + VAC set, −100 mmHg × 5 days or longer	Standard dressing	Sublay, intraperitoneal	Synthetic or biological	1) 3) 4) 5) 6)
Deldar et al. (2022) [14]	Single center (USA)	Retrospective	55.0:52.9	36.4:36.2	114 (NPWT, 57)	6.64 months	VAC or PREVENA for 5–7 days	Standard dressing	Onlay, sublay, intraperitoneal	Synthetic, biological or composition	1) 2) 3) 4) 5) 7)
Diaconu et al. (2020) [15]	Single center (USA)	Retrospective	58.0:50.0	41.0:40.0	104 (NPWT, 62)	190 days	PREVENA, −125 mmHg × 5 days	Standard dressing	Onlay, inlay, sublay	No mesh, biological or synthetic	1) 2) 3) 4) 5) 6) 7)
Gassman et al. (2013) [16]	Multicenter (USA)	Retrospective	NR	NR	61 (NPWT, 29)	167 days	VAC, −125 mmHg × 7 days	Standard dressing	Intraperitoneal	Biological	1) 3) 4) 6) 7)
Hopkins et al. (2020) [17]	Single center (Canada)	Retrospective	61.0:59.0	31:31	114 (NPWT, 41)	180 days	PREVENA, −125 mmHg × 5–7 days	Standard dressing	Intraperitoneal, extraperitoneal, onlay	Synthetic, biological, composition	1) 2) 3) 4) 5) 7)
Leuchter et al. (2021) [18]	Single center (Germany)	Retrospective	56.6:63.1	31.3:30.6	108 (NPWT, 54)	38.5 months	PREVENA, −125 mmHg × 7 days	Standard dressing	Sublay	NR	1) 2) 3) 4) 5) 6) 7)
Licari et al. (2020) [19]	Single center (Italy)	Retrospective	73.3:75	28.0:26.0	180 (NPWT, 70)	90 days	PREVENA, −125 mmHg × 7 days	Standard dressing	Sublay, intraperitoneal	Synthetic	1) 2) 3) 5)
Mondal et al. (2022) [20]	Single center (India)	RCT	47.0:43.0	23.9:23.8	64 (NPWT, 30)	4 weeks	Home-made wound dressing, −125 mmHg × 5 days	Gauze dressing	Onlay	Synthetic	1) 3)
Olona et al. (2014) [21]	Single center (Spain)	Retrospective	71.0:62.0	28.1:30.08	42 (NPWT, 5)	NR	PREVENA, −125 mmHg × 7 days	Standard dressing	Onlay	Synthetic	1) 3) 4)
Pauli et al. (2013) [22]	Single center (USA)	Retrospective	56.3:55.4	33.5:34.8	119 (NPWT, 49)	30 days	Home-made wound dressing, −75 mmHg × 5 days or discharge	Sterile dry gauze adhesive dressing	Sublay	Synthetic or biological	1)
Seaman et al. (2021) [23]	Single center (USA)	Retrospective	58.0:56.0	33.0:32.0	258 (NPWT, 159)	30 days	NR	Standard dressing	NR	NR	1) 2) 3) 4) 5) 6) 7)
Soares et al. (2015) [24]	Single center (USA)	Retrospective	56.0:56.0	33.5:32.3	199 (NPWT, 115)	9 months	Home-made wound dressing, −125 mmHg × 3 days	Standard dressing	Onlay, preperitoneal, onlay, preperitoneal	Synthetic and biological	1) 2) 3) 5) 7)
Wang et al. (2021) [25]	Single center (Australia)	Retrospective	58.0:65.0	36.4:39.2	35 (NPWT, 23)	NR	PREVENA, −125 mmHg × 7 days	Standard dressing	Sublay	Biological, composite, or polypropylene	1) 3) 5)

Remark: NR: not reported, outcomes: 1) SSI; 2) SSO; 3) seroma; 4) wound hematoma; 5) wound dehiscence; 6) skin necrosis; 7) hernia recurrence

### Assessment of the included studies

**Fig. 2:**
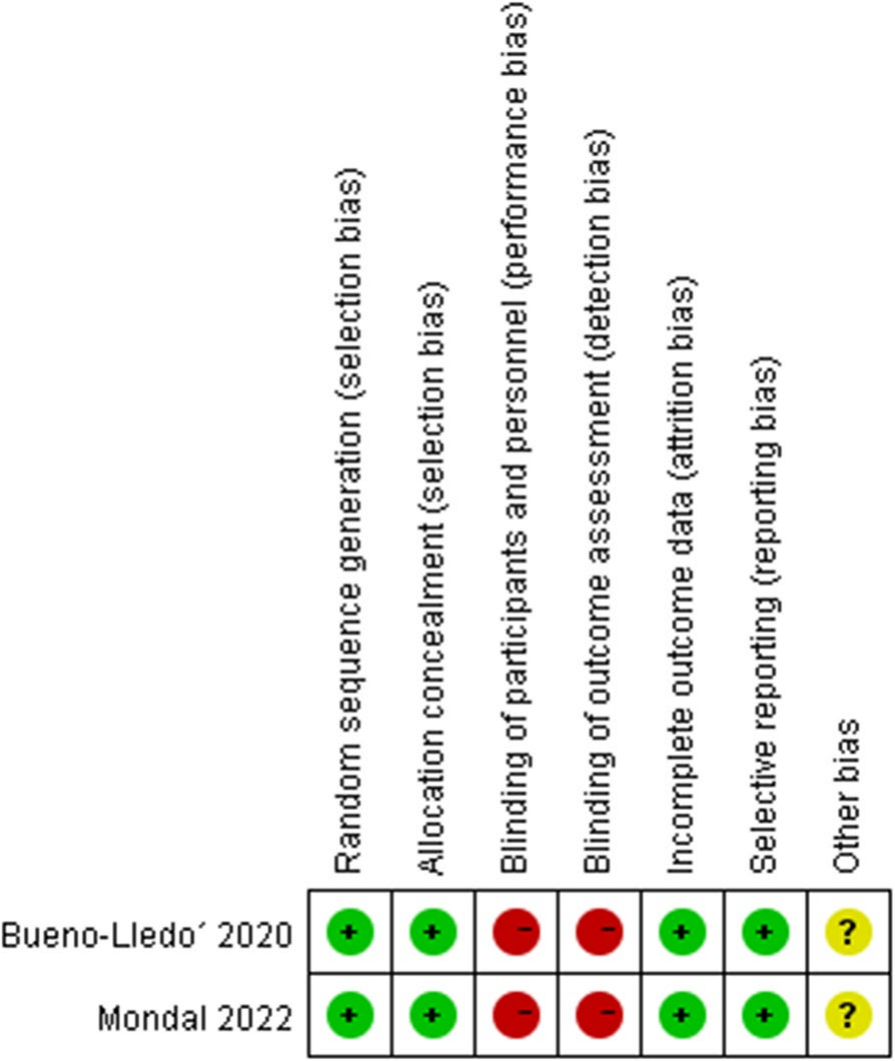
Risk of bias assessment for the included RCTs

According to the Newcastle-Ottawa Scale for retrospective cohort study, 9 cohort studies were ranked as high quality (scores≥7) and 4 studies as medium quality (Table. 2). Only Bueno-Lledó and Mondal conducted RCTs, both of which had detailed descriptions of randomization and data collection except blinding (Fig. 2).

**Table 2:** Results of quality assessment using the Newcastle-Ottawa Scale for the retrospective cohort study

Study	Selection				Comparability	Exposure			Scores
	Adequate definition of cases	Representativeness of the cases	Selection of controls	Definition of controls	Control for important factors	Ascertainment of exposure	Same method of ascertainment for cases and controls	Non-response rate	Scores
Conde´-Green 2013 [12]	★	★	★	★	☆☆	★	★	☆	6
de Vries 2017 [13]	★	★	★	★	★☆	★	★	☆	7
Deldar 2022 [14]	★	★	★	★	★★	★	★	☆	8
Diaconu 2020 [15]	★	★	★	★	★☆	★	★	☆	7
Gassman 2013 [16]	★	★	★	★	★☆	★	☆	☆	6
Hopkins 2020 [17]	★	★	★	★	★★	★	★	★	9
Leuchter 2021 [18]	★	★	★	★	★★	★	★	☆	8
Licari 2020 [19]	★	★	★	★	★☆	★	★	☆	7
Olona 2014 [21]	☆	★	★	★	★☆	★	☆	☆	5
Pauli 2013 [22]	★	★	★	★	★★	★	★	☆	8
Seaman 2021 [23]	★	★	★	★	★☆	★	★	☆	7
Soares 2015 [24]	★	★	★	★	★★	★	★	☆	8
Wang 2021 [25]	☆	★	★	★	☆☆	★	☆	☆	4

### SSO

**Fig. 3:**
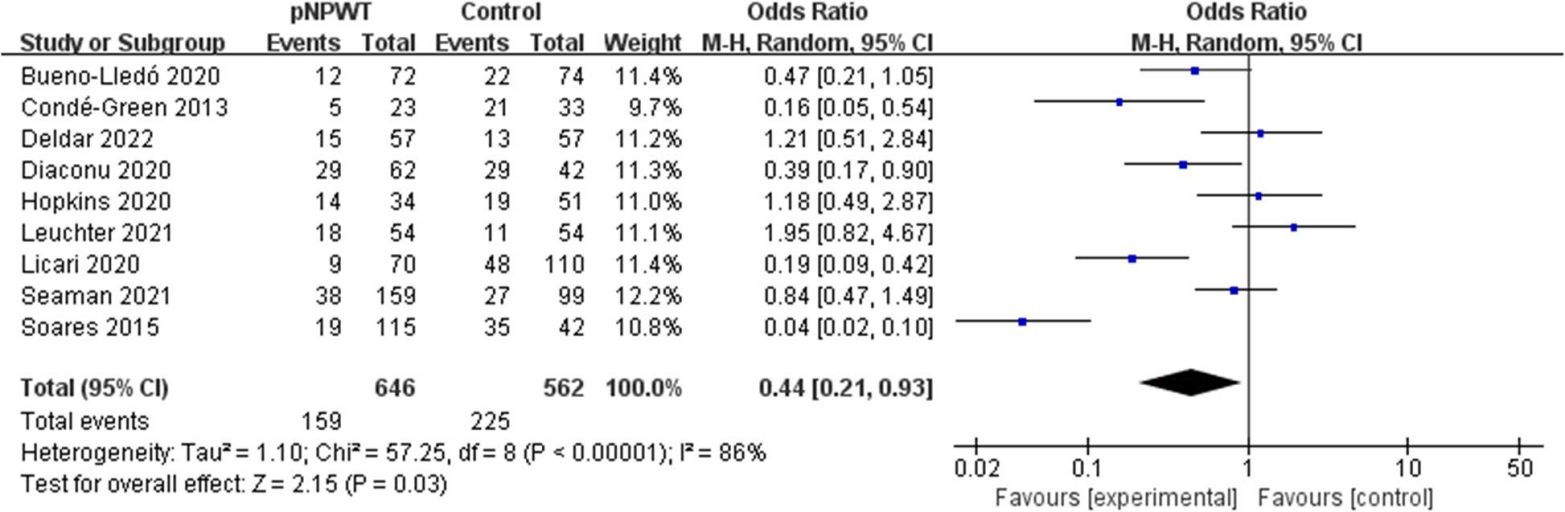
Meta-analysis of SSO between the pNPWT and control group

In the SSO analysis, a total of 8 articles were included and 1208 patients were involved. A random-effects model was used in the analysis considering the high heterogeneity (I^2^ = 86%, *P* < 0.001). The rate of SSO was significantly different in patients who received pNPWT compared with those who received standard dressing. About 24.6% (159/646) of patients who underwent pNPWT reported SSO at follow-up compared to 40.0% (225/562) who received standard dressings (pooled OR = 0.44; 95% confidence interval [CI] = 0.21–0.93; *P* = 0.03; Fig. 3). Subgroup analysis revealed differences in dressings and article quality as possible sources of heterogeneity (*P* ≤ 0.05). Due to the low number of studies, Egger’s test was used to evaluate publication bias and the results revealed no definite publication bias (*P* > 0.05).

### SSI

**Fig. 4:**
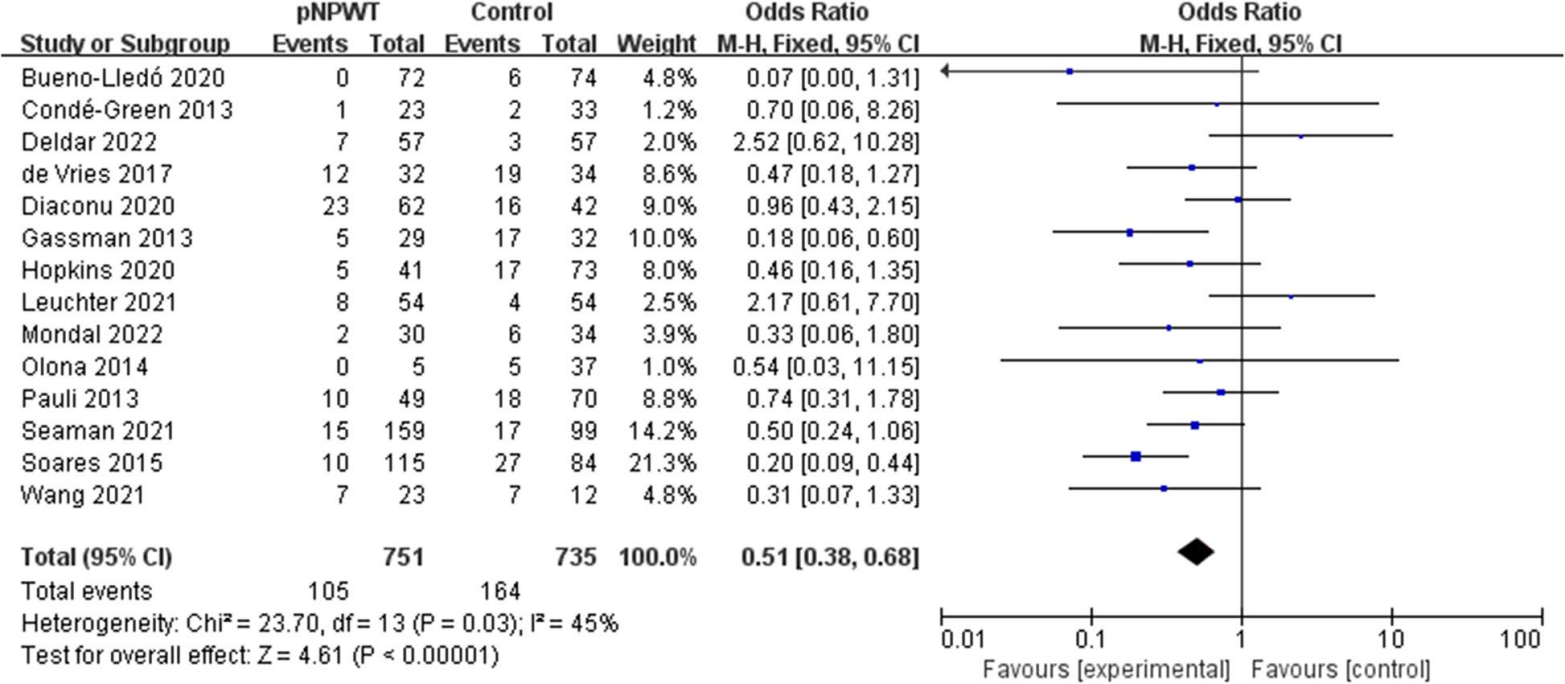
Meta-analysis of SSI between the pNPWT and control group

After pooling all included studies reporting SSI rates (14 studies, 1486 patients), we found that 11.4% (76 of 665) of the patients who received pNPWT developed SSI compared with 14.0% (105 of 751) of those who received standard dressing; pNPWT could effectively reduce the SSI rate (pooled OR = 0.51; 95% CI = 0.38–0.68; *P* < 0.001; Fig. 4). Medium heterogeneity in this analysis (I^2^ = 45%) and the random effects model were selected.

### Seroma and hematoma

**Fig. 5:**
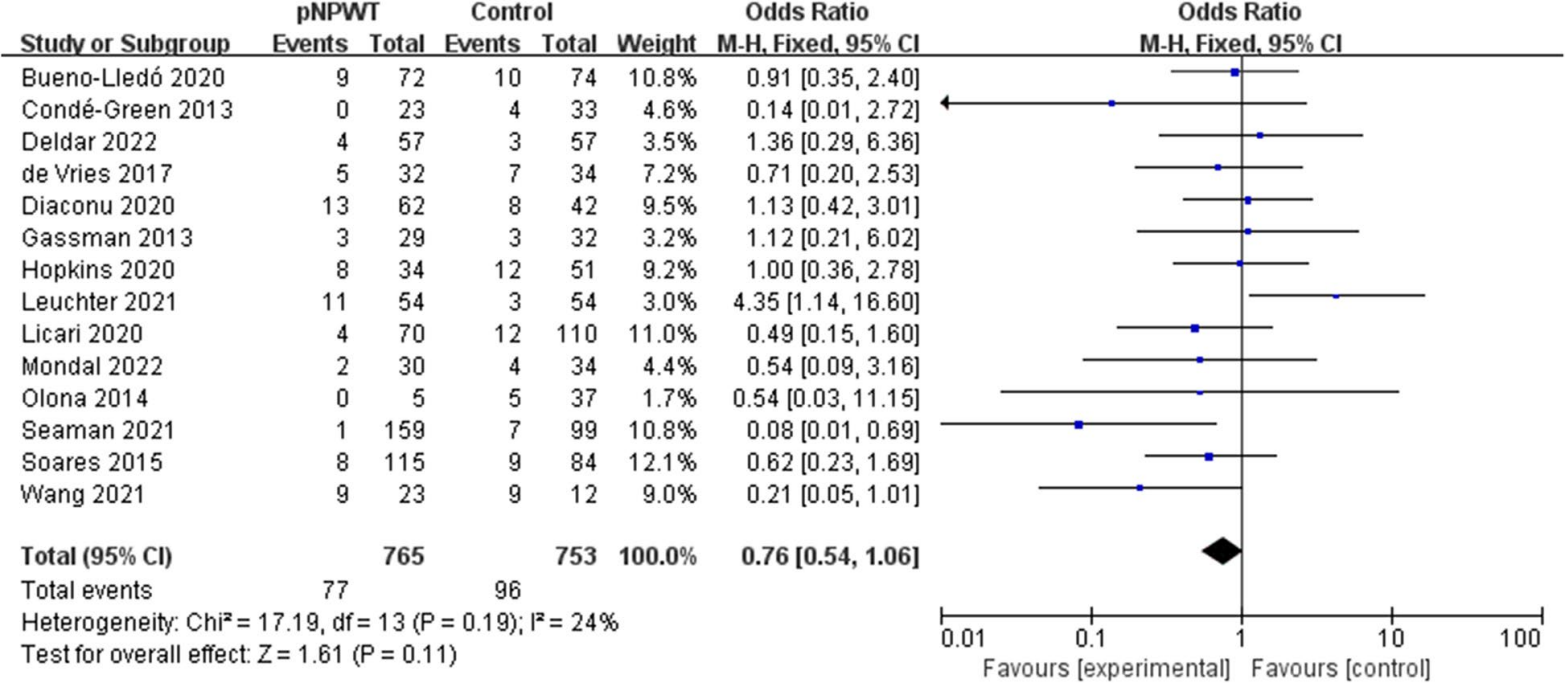
Meta-analysis of seroma between the pNPWT and control group

**Fig. 6:**
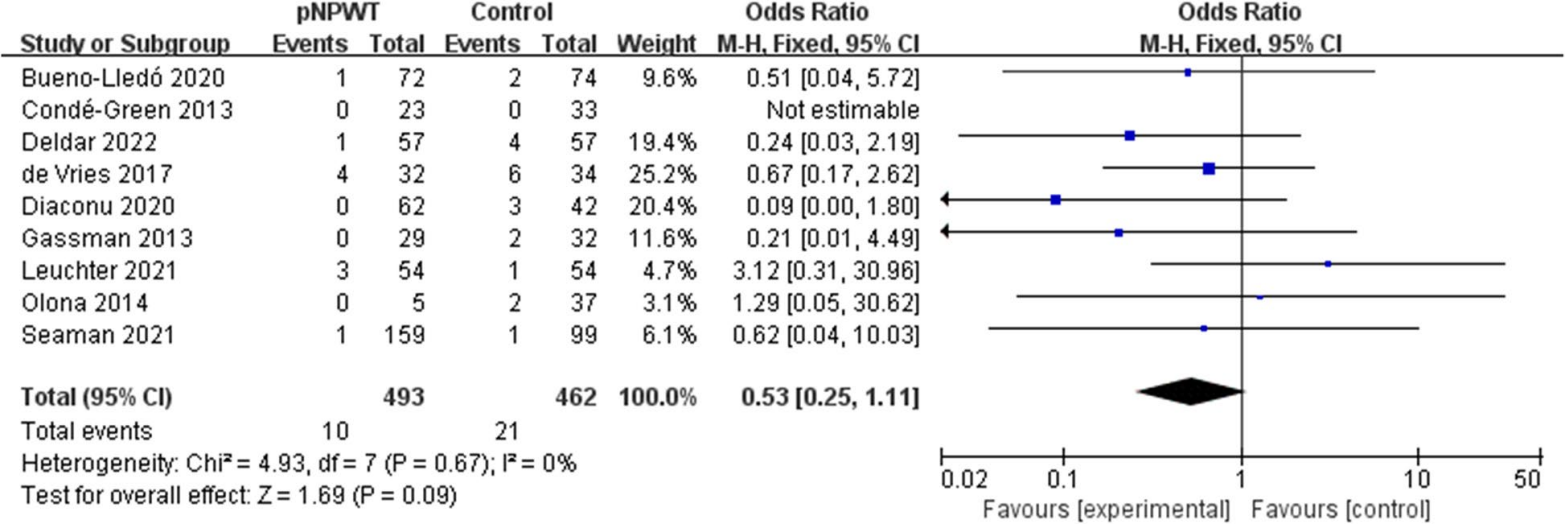
Meta-analysis of hematoma between the pNPWT and control group

Seroma and hematoma were used as outcome measures in 14 and 9 studies, respectively. The fixed-effects model was used for the results because none suggested high heterogeneity (seroma: I^2^ = 24%; hematoma: I^2^ = 0%; Figs. 5 and 6). There was no significant difference between the pNPWT group and control group (standard dressing) in seroma (OR = 0.76; 95% CI = 0.54–1.06; *P* = 0.11) and hematoma (OR = 0.53; 95% CI = 0.25–1.11; *P* = 0.09).

### Wound dehiscence and skin necrosis

**Fig. 7:**
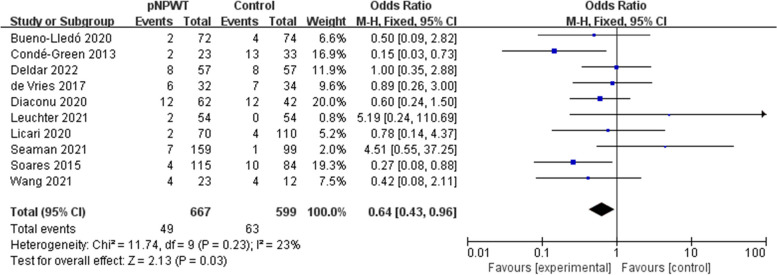
Meta-analysis of wound dehiscence between pNPWT and control group

**Fig. 8:**
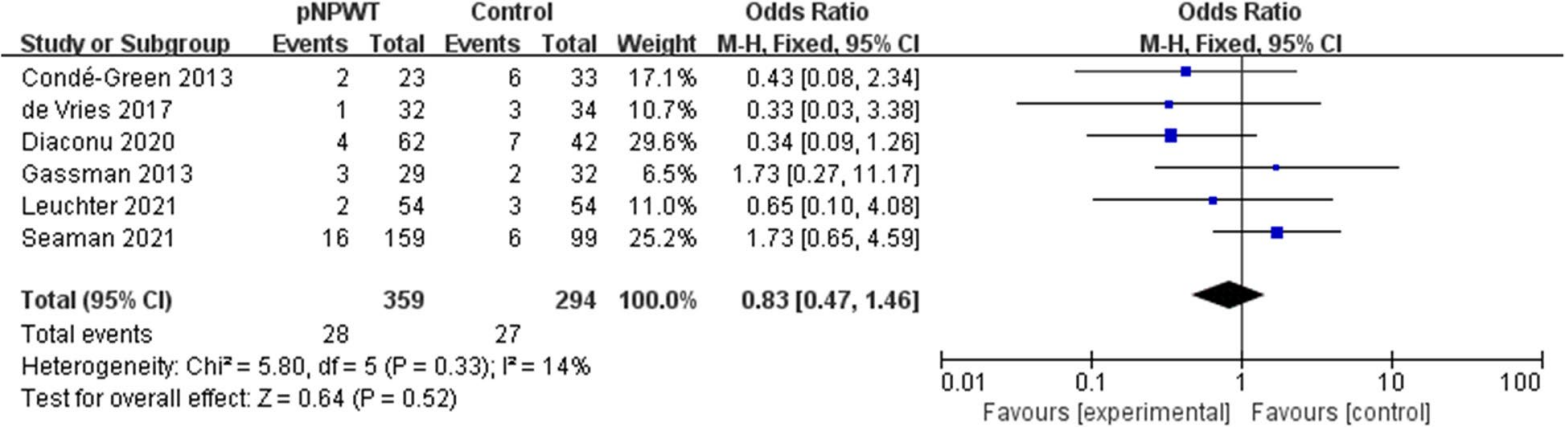
Meta-analysis of skin necrosis between the pNPWT and control group

Eleven and 5 studies included wound dehiscence and skin necrosis as outcome measures, respectively. Heterogeneity was ascertained as low (wound dehiscence: I^2^ = 23%; skin necrosis: I^2^ = 14%; Fig. 7 and Fig. 8). Prophylactic NPWT appeared to be protective against wound dehiscence compared to standard dressing (OR = 0.64; 95% CI = 0. 43–0.96; *P* = 0.03). The occurrence of skin necrosis was evident in 7.3% (28/359) of patients after pNPWT compared to 9.2% (27/294) of patients after standard dressing (*P* = 0.52).

### Hernia recurrence

**Fig. 9:**
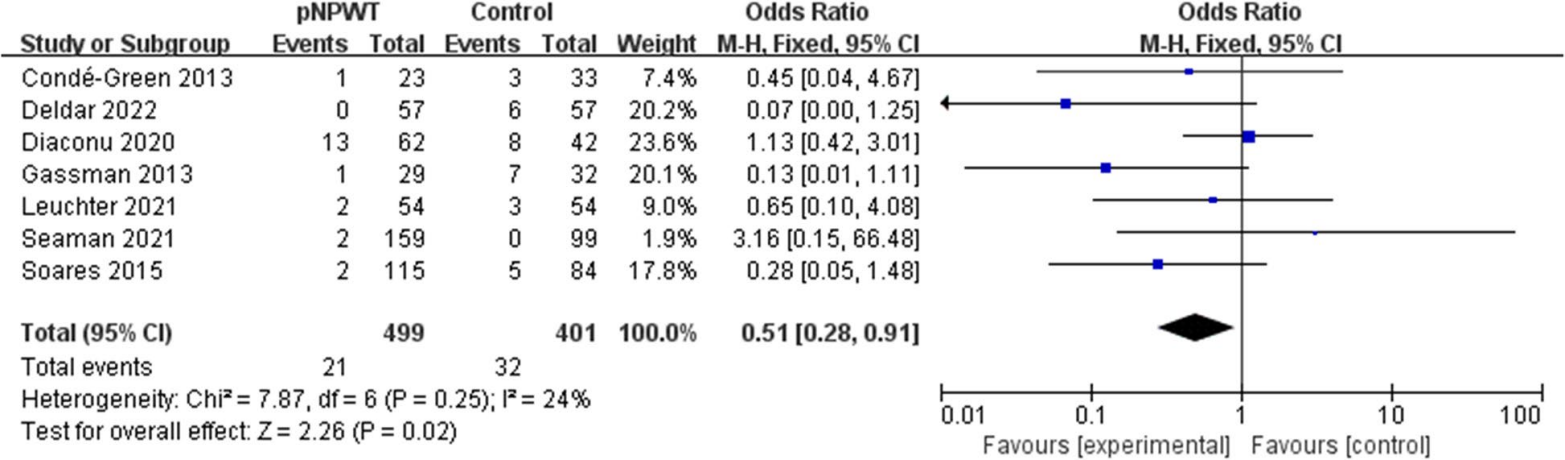
Meta-analysis of hernia recurrence between the pNPWT and control group

Seven studies included hernia recurrence rate as an outcome measure. A fixed-effect model was used in the analysis considering low heterogeneity (I^2^ = 24%, *P* = 0.25). Patients receiving pNPWT had a significantly decreased risk of hernia recurrence compared with standard dressing; 4.7% (21/449) of patients who received pNPWT reported SSO at follow-up compared to 8.0% (32/401) of those who received standard dressings (pooled OR = 0.51; 95% CI = 0.28–0.91; *P* = 0.02; Fig. 9).

## Discussion

VHR is a common surgical procedure, with more than 400,000 surgeries performed in the USA alone annually [26]. Laparoscopic VHR is becoming a general trend due to its lower perioperative complication rate, shorter hospital stays, and lower postoperative readmission rate [27]. Regardless of the surgical method (whether open or endoscopic), postoperative complications remain a challenge for surgeons. Without a doubt, the outcomes could be extremely complex, leading to increased medical costs to patients and hospitals.

NPWT is commonly used to treat open wounds in the abdomen in combination with lavage, debridement, and bowel resection as indicated [28]. Since its introduction in 1997, NPWT has been shown to be effective in both open and postoperative closed wounds [29]. However, the efficacy of pNPWT in wound healing after OVHR is unclear and data are contradictory in the literature [30]. In the earliest RCT in 2020 including 150 patients, Bueno-Lledó et al. showed that the incidence of SSO in the control group (standard dressing; 22/74, 29.8%) was significantly higher than that in the NPWT group (12/72, 16.6%, *P* < 0.05) after 30 days of postoperative follow-up [31]. Deldar et al. found that NPWT had no effect on the prevention of SSO (23.2% vs 26.3%, *P* = 0.663) [23]. In addition, the two RCTs to date have different results in the SSI study [11]. The existing related studies were mostly retrospective cohort studies or case series. We analyzed 15 articles and the results of the meta-analysis from all included studies showed that pNPWT significantly reduced the risk of SSO, SSI, wound dehiscence, and the rate of hernia recurrence (*P* < 0.05). Meanwhile, there was no significant difference in seroma, hematoma, and skin necrosis with pNPWT compared to standard wound dressings.

It is worth noting that with the addition of a recent RCT, we obtained the same results for SSO as in the previously published meta-analysis articles [32]. Nevertheless, a high degree of heterogeneity (I^2^ = 87%) is seen in the SSO results. Further subgroup analysis suggested that the heterogeneity stemmed from differences in dressings and the quality of studies. The probable reason for the difference in the test results is variations in the diagnostic methods and surgeon-dependent definition of SSO and operation level. However, due to the small number of studies, we did not use the traditional funnel plot for analysis of publication bias. Also, Egger’s test results revealed no publication bias in SSO. No high heterogeneity was found in the analysis of SSI, seroma, hematoma, wound dehiscence, skin necrosis, and hernia recurrence.

NPWT has been shown to have a crucial role in scar healing following OVHR [33]. NPWT can enhance wound drainage, eliminate bacterial byproducts, discharge secretions efficiently, and clear necrotic tissue [34]. Under the mechanical traction of negative pressure, the differential pressure between the inner and outer capillaries and the endothelial cell space of lymphatic capillaries increases, resulting in elevated levels of blood supply and lymphatic reflux [35–37]. Compared with conventional dressings, a negative pressure provides effective and sustained support for local wound circulation, thereby reducing the risk of seroma and hematoma [11, 38]. Furthermore, continuous negative pressure extracts interstitial fluid from the wound, fosters growth of capillaries and granulation tissue, improves blood circulation, and accelerates scar healing [39, 40]. Through these mechanisms, NPWT lowers wound healing time and enhances scar recovery significantly. Additional studies are required to further understand the mechanism of NPWT in OVHR.

However, NPWT may entail certain complications, such as skin blisters [33], which typically resolve spontaneously within approximately a week [41]. Improper usage of NPWT has the potential to result in more severe complications, including skin necrosis, bleeding, and allergic reactions [42, 43]. It is imperative to discontinue NPWT in case these complications occur. Nevertheless, studies indicate that there is no noteworthy increase in the incidence of wound-related adverse events associated with NPWT when compared to standard dressings [44]. Appropriate preventive measures may help mitigate adverse events [45].

There are some limitations of this meta-analysis. First, most studies involved in this meta-analysis were retrospective and only two RCTs were included, which may introduce a selection bias in the process [46]. Second, the definition of SSO was different among articles. Most authors defined SSO as surgical site infection (SSI), wound dehiscence, skin necrosis, seroma, and hematoma. Seaman et al. believed that enterocutaneous fistula, mesh infection, hernia recurrence, and/or 30-day bulge are considered when detecting SSO [30], which is bound to make a difference to the SSO results. Third, there is a lack of standard diagnostic criteria and follow-up time for complications occurring in these studies. For example, seroma was diagnosed clinically or using ultrasound, with significantly different incidences. Further, the shortest follow-up period was 1 month, while the longest follow-up period was 38.5 months. Fourth, most of the articles did not report any recurrent hernias or emergency hernias, which might also affect the occurrence of SSO. Finally, there was no standard for the defect range in the included articles. Only a few studies used the defect size or European Hernia Society (EHS) classification. The guidelines of the International Endohernia Society (IEHS) also clearly state that abdominal wall defects are closely related to postoperative SSO and the recurrence of hernia [47]. This also requires newer studies to further verify how large a defect is suitable for placing pNPWT.

Certainly, if only the device cost is considered, the cost of closing surgical wounds with NPWT is higher than that with traditional dressings. In the study by Clare et al., after accounting for postoperative complications and hospitalization costs, a cost-benefit analysis showed an estimated total cost reduction of €170,944.00 in 100 people treated with closed incision negative pressure therapy (ciNPT) compared to standard wound care [15]. Svensson-Björk et al. concluded that NPWT can reduce the incidence of SSI, which was more cost-effective than standard dressings; the average cost increase was €1853 after following patients for 90 days following open inguinal vascular surgery [48]. Considering the cost of analgesics, antibiotics, hospitalization, and morbidity, Condon et al. concluded that the cost of satisfactory healing in people who received NPWT was lower compared to conventional dressing for the treatment of wounds due to diabetic foot [19].

## Conclusions

Our review concludes that the use of pNPWT after OVHR can reduce the incidence of SSO and SSI, wound dehiscence, and hernia recurrence simultaneously. Prophylactic NPWT has no obvious effect on other complications, such as seroma, hematoma, and skin necrosis. However, the large heterogeneity of studies regarding SSO is a limitation of the review, possibly due to differences in article quality and NPWT techniques. There is an urgent need for higher quality, better-designed RCTs with consistent standards to further verify the efficacy of pNPWT following OVHR.

### Supplementary Information

**Additional file 1.**

**Additional file 2.**

## Data Availability

All authors have agreed to make provisions for all materials described in this manuscript including relevant raw data to be freely available to any researchers.

## Abbreviations

VHR: Ventral hernia repair
SSO: Surgical site occurrence
NPWT: Negative pressure wound therapy
OVHR: Open ventral hernia repair
RCTs: Randomized controlled trials
OR: Odds ratio
CI: Confidence interval
SSI: Surgical site infection
VH: Ventral hernia
BMI: Body mass index
pNPWT: Prophylactic negative pressure wound therapy
VAC: Vacuum-assisted closure
PRISMA: Preferred Reporting Items for Systematic Reviews and Meta-analyses
EHS: European Hernia Society
IEHS: International Endohernia Society
ciNPT: Closed incision negative pressure therapy
