# Supplementary figures and images for: Efficacy of prophylactic negative pressure wound therapy after open ventral hernia repair: a systematic review meta-analysis
Source: BMC Surg. 2023 Dec 11;23:374. doi: 10.1186/s12893-023-02280-4 (PMC10712064; doi:10.1186/s12893-023-02280-4)

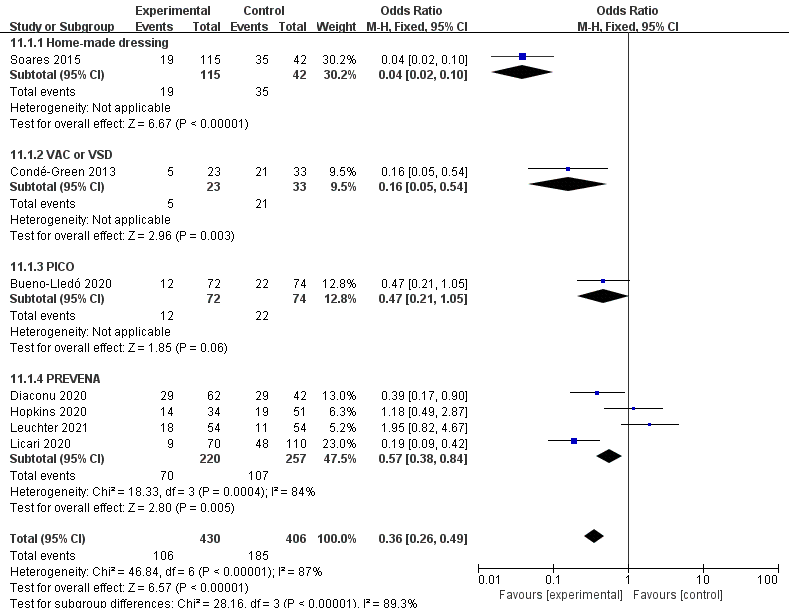

Supplement: Supplementary file 1 — Additional file 1. [file 12893_2023_2280_MOESM1_ESM.tif]

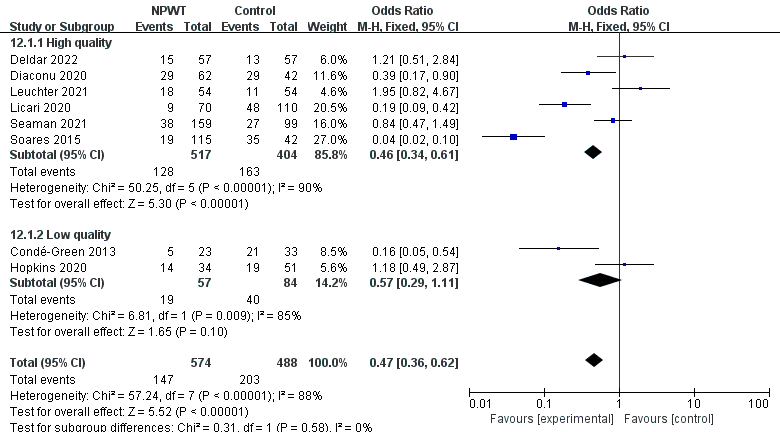

Supplement: Supplementary file 2 — Additional file 2. [file 12893_2023_2280_MOESM2_ESM.tif]
